# Put your publication money where your mouth is

**DOI:** 10.1093/braincomms/fcad220

**Published:** 2023-08-31

**Authors:** Tara L Spires-Jones, David Belin

**Affiliations:** Edinburgh, UK; Cambridge, UK

## Abstract

Two members of our Editorial Board discuss how the proceeds from article processing charges from *Brain Communications* and our sister journal *Brain* are put back into the translational neuroscience community.

## Graphical Abstract

**Graphical Abstract:**
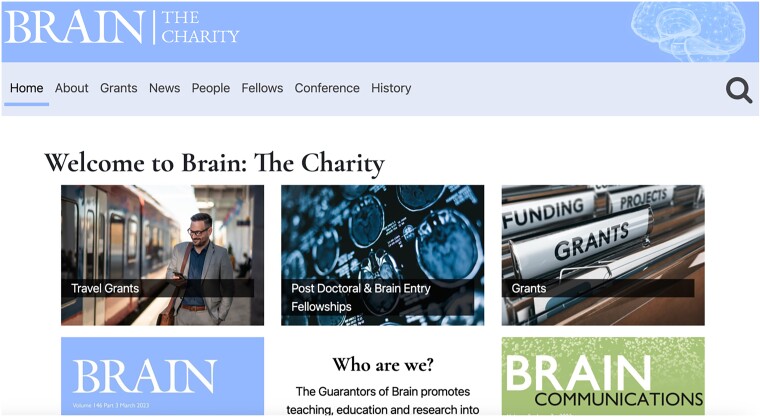

Welcome to Volume 5, Issue 5 of *Brain Communications*. In this editorial, we will try to convince you that publishing in academic-led, community-oriented journals like ours is a better use of your hard-earned grant money than publishing in for-profit journals. Where to send papers for publication can be a difficult choice and one that often leads to heated debates amongst authors. We all want to find the right ‘home’ for our research, to publish it in journals visible to our peers. For many, this is synonymous with high-impact journals, especially since publications in high ‘impact’ journals are still considered by some institutions to be important for promotions and grant success.

However, the impact of the paper is not determined by the impact factor of the journal in which it is published, but rather, by whether the science it presents is impactful and whether the right audience is targeted.

Perhaps, another important factor to consider in choosing where to send papers, which may not always come to mind, is where the money earned by the journal goes. For the society or charity-owned journals like ours, the surplus funds raised beyond the costs associated with publishing are put back into the scientific community. In our case, The Guarantors of Brain charity uses money raised by *Brain* and *Brain Communications* to support fellowships, meetings, and travel grants to attend conferences or to do pro-bono work in low-income countries (see https://guarantorsofbrain.org/). The Guarantors of Brain have funded over 50 fellowships in the past 4 years to support both clinical and non-clinical neuroscientists early in their careers (see https://guarantorsofbrain.org/brain-fellows/). In a very satisfying full-circle, several of these fellows have published their work in our journals, see for example Sheybani *et al*. ^1^ and Ruffle *et al*.^2^

Similar society journals in the neuroscience space that we are also fans of are the *European Journal of Neuroscience* which supports the Federation of European Neuroscience, *Brain and Neuroscience Advances* which supports the British Neuroscience Association, the *Journal of Neuroscience* which supports the US Society for Neuroscience, and *Science* which supports the American Association for the Advancement of Science.

While all charity journals support the aims and values of their societies, some still use publishing partners that make a profit like Wiley and Elsevier. Elsevier and Springer-Nature (the publisher of the *Nature* journals) have come under fire for charging what some scientists see as exorbitant fees for publications to raise their already high-profit margins.^3,4^ In our case, our publishing partner, Oxford University Press, is also a not-for-profit department of the University of Oxford, whose mission is to further excellence in research, scholarship, and education (see http://global.oup.com/).

We are of the view that such considerations, which are key to building a more inclusive scientific community, should take precedence over that of the impact factor of the journal. In our view, it is the responsibility of all of us when acting on hiring and promotions committees and grant panels to deflate the undue weight put on the impact factor of the journal where a work has been published and add more value to the strength of the work instead. Avid readers of *Brain Communications* may remember we wrote our thoughts on the use of impact factor as a metric for papers in an editorial last year.^5^ In the meantime, our journal has been bestowed an impact factor by Clarivate. We are proud that our papers are being read and cited by the community, but we are equally proud of our ethos to value replication studies and de-emphasize the need for novelty, so we are not widely promoting our impact factor, although it is on our website if you want to see it.

The cover image of this issue shows a piece of donated cortical brain tissue received at autopsy before processing for high-resolution imaging and molecular studies in Professor Tara Spires-Jones’ lab. This heart-shaped piece of tissue reminds authors of the generosity of our brain donors and their families who made the research possible.
